# Genetic structure analysis of *Glycyrrhiza* species and identification of SNP-loci associated with secondary metabolites in *G. glabra*

**DOI:** 10.1186/s12864-026-13361-y

**Published:** 2026-09-18

**Authors:** Ali Moghadam, Akbar Karami, Beate Kellert, Reza Haghi, Hassan Esmaeili, Axel Himmelbach, Lars-Gernot Otto

**Affiliations:** 1 Institute of Biotechnology, Shiraz University, P.O.Box: 71441-13131, Shiraz, Iran; 2 Department of Horticultural Science, Shiraz University, P.O.Box: 71441-13131, Shiraz, Iran; 3 Department Plant Breeding Research, Leibniz Institute of Plant Genetics and Crop Plant Research (IPK), Quantitative Genetics Research Group, Corrensstrasse 3, 06466 Seeland OT Gatersleben, Germany; 4 Department Genebank, Leibniz Institute of Plant Genetics and Crop Plant Research (IPK), Domestication Genomics Research Group, Corrensstrasse 3, 06466 Seeland OT Gatersleben, Germany; 5 Department Agriculture, Medicinal Plants and Drugs Research Institute, Shahid Beheshti University, Tehran, 1983969411 Iran; 6 Department Genebank,, Genomics of Genetic Resources Research Group, Leibniz Institute of Plant Genetics and Crop Plant Research (IPK), Corrensstrasse 3, 06466 Seeland OT Gatersleben, Germany

**Keywords:** Licorice, *Glycyrrhiza*, Medicinal plant, Genetic diversity, Genotyping-by-sequencing, GWAS

## Abstract

**Background:**

*Glycyrrhiza* spp. are medicinal legumes that produce a wide array of pharmacologically active secondary metabolites. This study generated a genomic resource by genotyping-by-sequencing 175 *Glycyrrhiza* accessions and populations to (1) characterize their genetic structure and assess population relationships, and (2) perform an association mapping for three medically important traits within *G. glabra*.

**Results:**

We identified 38,393 high-quality single-nucleotide polymorphisms (SNPs). Genetic structure analysis revealed that *G. glabra* is mostly distinct from the other species, and identified one group of 66 plants from 33 Iranian *G. glabra* origins with moderate genetic differentiation. The association mapping identified in total 20 highly significant SNPs for three traits: 15 for glabridin in the cork layer of the root, of which 3 were also associated with glabridin in the fleshy texture of the root, and 5 for liquiritigenin in the cork layer of the root. Glabridin and liquiritigenin are described to be involved in biotic and abiotic stress responses. Candidate genes include: (1) MYB6 – a transcription factor that activates flavonoid-biosynthetic genes. (2) OCTOPUS-like – a regulator of primary root protophloem differentiation and root architecture. (3) WRKY transcription factors – modulators of phenylpropanoid, alkaloid and terpene pathways. (4) Aminodeoxychorismate synthase.

**Conclusions:**

Our study provided a comprehensive understanding of the genetic diversity of *Glycyrrhiza* spp. We identified genetic markers associated with important metabolites of the root, i.e. the flavonoids glabridin and liquiritigenin. These findings will facilitate the exploitation of genetic resources and inform future breeding programs, ultimately contributing to the development of improved *Glycyrrhiza* spp. varieties with enhanced medicinal properties.

**Supplementary Information:**

The online version contains supplementary material available at https://doi.org/10.1186/s12864-026-13361-y.

## Background

Licorice is one of the most widely used medicinal plants in the world, with a history spanning thousands of years in traditional medicine systems [1]. The genus of *Glycyrrhiza* (Fabaceae) is described to consist of up to approx. 30 species [2, 3], of which *Glycyrrhiza glabra, G. uralensis, G. inflata, G. eurycarpa* are generally recognized as licorice for their sweet taste [4]. The global market for licorice derivatives, particularly glycyrrhizin and glabridin, has been growing steadily, driven by their extensive applications in the pharmaceutical, food, and cosmetic industries [5, 6]. Glycyrrhizin is found exclusively in the woody parts of the thickening roots and stolons, whereas a large amount of glabridin, a prenylated flavonoid, was detected exclusively in the cork layer and the decayed part of the thickening roots [7]. Current estimates place it at around USD 2.6 billion in 2025 and projected to surpass USD 4.0 billion by 2031 [8]. These compounds have shown several biological activities, such as anti-inflammatory, anti-cancerous, anti-ulcer, anti-spasmodic, anti-allergic and anti-viral properties [9].

The licorice species are herbaceous, perennial plants which possesses stems of 1–1.7 m in length, composite leaves, violet to purple flowers, nut-brown fruits of 2–3 cm in length. The *Glycyrrhiza* species have typically a chromosome number of 2n = 16, with exceptions being reported for *G. asymmetrica* with 14 chromosomes [4], and *G. pallidiflora* with reported 16 and 18 chromosomes [10]. The present *Glycyrrhiza* species are likely to be autogamous species [11]. The genus *Glycyrrhiza* exhibits a broad global distribution across Europe, Asia, North Africa, Australia, and temperate regions of North and South America [2] with the center of diversity being located in Eurasia, particularly in Central Asia. According to Flora Iranica, the three species *G. glabra*, *G. echinata* and *G. aspera* have been identified in Iran [12]. *Glycyrrhiza glabra* is one of the important species threatened by overharvesting worldwide and is protected in many habitats of Iran.

Population genetics studies are essential for characterizing the genetic structure and diversity within and between populations, which is a prerequisite for effective conservation strategies and for the domestication and molecular breeding of high-yielding, high-quality genotypes [13]. Furthermore, such studies can identify genomic regions under natural selection that are linked to key traits like the biosynthesis of valuable secondary metabolites (e.g., glycyrrhizin and glabridin), providing crucial insights for genetic improvement and sustainable utilization [14].

Previous genetic studies on *Glycyrrhiza* spp. have predominantly relied on traditional molecular markers with limited genomic coverage like SSRs, RFLPs, AFLPs (simple sequence repeat; restriction fragment length polymorphism; amplified fragment length polymorphism) [15–17]. High polymorphism and moderate genetic differentiation indicate shared ancestry with regional divergence [17]. While these marker systems have provided valuable baseline information, they offer limited datapoints for population structure analysis and limited resolution for dissecting the genetic architecture underlying key medicinal traits. The specific genes and regulatory networks controlling the biosynthesis of major bioactive compounds, including glycyrrhizin, glabridin, and liquiritigenin, remain largely unknown [18, 19].

Next-generation sequencing (NGS) methods have been successfully used to analyze and exploit genetic resources and genes in crop breeding, including the major crops rapeseed, soybean, barley, or maize [20, 21]. The NGS method genotyping-by-sequencing (GBS) has been widely used [e.g. [22, 23], is highly reproducible, and reduces genome complexity through restriction enzymes, which reduces costs [24]. The recent advances in next-generation sequencing have begun to address these limitations in *Glycyrrhiza*: chromosome-level reference genomes for *G. uralensis*, *G. inflata*, and *G. glabra* have been assembled [11], and integrative metabolomic-transcriptomic analyses have identified candidate genes and transcription factors involved in characteristic flavonoid biosynthesis [18]. Furthermore, while whole-genome resequencing has revealed genetic differentiation among the three medicinal species [19], the functional implications of this divergence for secondary metabolite production remain poorly characterized. However, neither a comprehensive population structure analysis based upon a large number of markers nor a genome-wide association study (GWAS) linking specific genetic variants to the accumulation of key pharmaceutical metabolites in *Glycyrrhiza* spp. have yet been reported.

Thus, to address these gaps we genotyped a set of *Glycyrrhiza* spp. origins (varieties, populations, combinations) by GBS to identify high-quality single nucleotide polymorphisms (SNPs) at a genome-wide scale, to estimate genetic diversity, and to reveal the genetic structure in a genetic material collection. Phenotyping data for 13 traits comprising secondary metabolites in *G. glabra* were available [7, 15]. *G. glabra* contains various pharmaceutical active substances as secondary metabolites [25]. For the key root metabolites glabridin and liquiritigenin, we conducted a GWAS to identify associated SNPs and potential candidate genes. This work established a resource for marker-assisted breeding to enhance medicinal compound production in licorice. SNP markers can help breeders by eliminating the cost and time needed to conduct field trials with extensive phenotyping, and thus support to accelerate breeding programs for *Glycyrrhiza* spp.

By using a GBS approach, this study aimed to: (1) generate a genome-wide SNP panel for *Glycyrrhiza* spp., (2) estimate the genetic diversity and population structure across diverse origins of *Glycyrrhiza,* and (3) perform a GWAS to identify SNPs and candidate genes associated with the key metabolites glabridin and liquiritigenin in *G. glabra*, one of the important species threatened by overharvesting worldwide and protected in many habitats of Iran.

We hypothesized that a subset of SNPs identified via GBS can effectively predict the metabolic traits glabridin and liquiritigenin, thereby supporting future breeding programs aimed at producing high-value licorice varieties, e.g. through marker assisted selection.

## Materials and methods

### Plant materials

We analysed 175 *Glycyrrhiza* spp. from different regions (Table 1). In detail, 66 individuals originated from 22 regions throughout Iran, 105 individuals from 21 accessions were provided by the genebank of the IPK Gatersleben and 4 individuals from one accession by the University of Veterinary Medicine Vienna (Vetmeduni). The collection of the Iranian samples was carried out under national and scientific guidelines as described by Esmaeili et al. [7], and was based on International Standards for Sustainable Wild Collection of Medicinal and Aromatic Plants (ISSC-MAP) (Version 1.0), according to the Medicinal Plant Specialist Group Species Survival Commission of IUCN (The World Conservation Union). The permission to collect licorice plants from wild natural resources was obtained from the Iranian Organization of Forests and Rangeland (Government Organization). The collected Iranian plant material was formally identified by Hassan Esmaeili and Akbar Karami. Upon collection, the accessions were conserved in an ex-situ conservation field in the School of Agriculture, Shiraz University, Shiraz, Iran (Longitude: 52°35′17.98″E, Latitude: 29˚43′26.14″N and Altitude: 1798 (m)).

**Table 1:** Plant material with its source of supply

Region of population and individual code	ID	Genus	Species	Country and province	Longitude (E)	Latitude (N)	Altitude (m)
Ahar-2, −4, −6	I-1, I-2, I-3	Glycyrrhiza	glabra	Iran, East Azerbaijan	47°03′ 10.26″	38˚26′ 10.72″	1403
Baft-4, −7, −8	I-4, I-5, I-6	Glycyrrhiza	glabra	Iran, Kerman	56°27′ 57.6″	29˚15′ 7.1″	2241
Bajgah-3, −4	I-7, I-8	Glycyrrhiza	glabra	Iran, Farsi	52°35′ 17.98″	29˚43′ 26.14″	1798
Bardsir-2, −8	I-9, I-10	Glycyrrhiza	glabra	Iran, Kerman	56°15′ 21.94″	29˚52′ 40.41″	2338
Bojnord-3, −5, −6	I-11, I-12, I-13	Glycyrrhiza	glabra	Iran, North Khorasan	57°36′ 54.38″	37˚27′ 13.91″	970
Darab-2, −3, −7, −9	I-14 to I-17	Glycyrrhiza	glabra	Iran, Farsi	54°25′ 37.64″	28˚43′ 3.95″	1081
Eghlid-4, −7, −9	I-18, I-19, I-20	Glycyrrhiza	glabra	Iran, Farsi	52°29′37.9″	30˚44′ 30.8″	2319
GLY-Wien1, 2, 3, 4	I-21 to I-24	Glycyrrhiza	glabra	Vetmeduni Vienna**			
GLY10-1, −2, −3, −4, −5, −6	I-25 to I-30	Glycyrrhiza	echinata	genebank IPK Gatersleben			
GLY11-1, −2, −3, −4, −5, −6, −7, −8	I-31 to I-38	Glycyrrhiza	glabra	genebank IPK Gatersleben			
GLY12-1, −2, −3, −4	I-39 to I-42	Glycyrrhiza	pallidiflora	genebank IPK Gatersleben			
GLY13-1, −2, −3, −4	I-43 to I-46	Glycyrrhiza	uralensis	genebank IPK Gatersleben			
GLY14-1, −2, −3, −4	I-47 to I-50	Glycyrrhiza	pallidiflora	genebank IPK Gatersleben			
GLY15-1, −2,−3,−4	I-51 to I-54	Glycyrrhiza	glabra	genebank IPK Gatersleben			
GLY16-1, −3, −4	I-55, I-56, I-57	Glycyrrhiza	echinata	genebank IPK Gatersleben			
GLY17-1, −2, −3, −4, −5, −6	I-58 to I-63	Glycyrrhiza	echinata	genebank IPK Gatersleben			
GLY18-1, −2, −3	I-64, I-65, I-66	Glycyrrhiza	glabra	genebank IPK Gatersleben			
GLY20-1	I-67	Glycyrrhiza	aspera	genebank IPK Gatersleben			
GLY23-1, −2, −3, −4	I-68 to I-71	Glycyrrhiza	glabra	genebank IPK Gatersleben			
GLY24-1, −2, −3	I-72, I-73, I-74	Glycyrrhiza	glabra	genebank IPK Gatersleben			
GLY26-1, −2	I-75, I-76	Glycyrrhiza	echinata	genebank IPK Gatersleben			
GLY27-1, −2	I-77, I-78	Glycyrrhiza	uralensis	genebank IPK Gatersleben			
GLY3-1, −2, −3, −4, −5, −6, −7, −8	I-79 to I-86	Glycyrrhiza	echinata	genebank IPK Gatersleben			
GLY30-1, −2, −3, −4	I-87 to I-90	Glycyrrhiza	echinata	genebank IPK Gatersleben			
GLY4-1, −2, −3, −4, −5, −6	I-91 to I-96	Glycyrrhiza	echinata	genebank IPK Gatersleben			
GLY6-1, −2, −3, −4	I-97 to I-100	Glycyrrhiza	glabra	genebank IPK Gatersleben			
GLY7-1	I-101	Glycyrrhiza	glabra	genebank IPK Gatersleben			
GLY8-1, −2, −3, −4, −5, −6, −7, −8	I-102 to I-109	Glycyrrhiza	glabra	genebank IPK Gatersleben			
GLY9-1, −2	I-110, I-111	Glycyrrhiza	glabra	genebank IPK Gatersleben			
HajiAbad-3, −4, −5, −7, −8	I-112 to I-116	Glycyrrhiza	glabra	Iran, Hormozgan	55°44′ 38.4″	28˚17′ 12.6″	897
Ilam-1, −3, −4, −9	I-117 to I-120	Glycyrrhiza	glabra	Iran, Ilam	46°17′ 43.72″	33˚40′ 49.64″	1032
Kashmar-3, −8	I-121, I-122	Glycyrrhiza	glabra	Iran, Razavi Khorasan	58°27′ 51.07″	35˚23′ 59.70″	1632
Kazerun-10, −3, −5, −7, −8	I-123 to I-127	Glycyrrhiza	glabra	Iran, Farsi	51°50′ 26.5″	29˚33′ 59.4″	1258
Kermanshah-1, −2, −4, −5, −6, −7	I-128 to I-133	Glycyrrhiza	glabra	Iran, Kermanshah	46°59′ 21.37″	34˚23′ 05.91″	1371
Mahabad-1, −3, −4	I-134, I-135, I-136	Glycyrrhiza	glabra	Iran, West Azarbaijan	36˚48′ 12.68″	45°43′ 09.53″	1410
Marvest-1, −6	I-137, I-138	Glycyrrhiza	glabra	Iran, Yazd	54°13′ 51.9″	30˚26′ 59.8″	1542
MeshkinShahr-2, −4, −6, −9	I-139 to I-142	Glycyrrhiza	glabra	Iran, Ardabil	47°42′ 54.80″	38˚25′ 01.10″	1412
Naqade-4, −8, −9	I-143, I-144, I-145	Glycyrrhiza	glabra	Iran, West Azarbaijan	36˚55′ 22.52″	45°32′ 39.17″	1298
Piranshahr-10, −9	I-146, I-147	Glycyrrhiza	glabra	Iran, West Azarbaijan	36˚37′ 42.95″	45°07′ 54.92″	1492
Quchan-1, −2, −3, −4, −5, −7	I-148 to I-153	Glycyrrhiza	glabra	Iran, Razavi Khorasan	58°24′ 33.1″	37˚01′ 57.5″	1678
Rabt-1, −7	I-154, I-155	Glycyrrhiza	glabra	Iran, West Azarbaijan	36˚12′ 41.35″	45°31′ 54.68″	1075
Saqez-5, −6, −8	I-156, I-157, I-158	Glycyrrhiza	glabra	Iran, Kordistan	36˚15′ 20.55″	46°12′ 55.33″	1683
Sepidan-1, −3, −5	I-159, I-160, I-161	Glycyrrhiza	glabra	Iran, Farsi	52°00′ 41.5"	30˚13′ 21.5"	2157
ShahreBabak-1, −2, −3	I-162, I-163, I-164	Glycyrrhiza	glabra	Iran, Kerman	30˚08′ 08.99″	55°09′ 07.31″	1874
Shahreza-2	I-165	Glycyrrhiza	glabra	Iran, Isfahan	51°54′ 23.52″	32˚02′ 32.94″	1786
Sirjan-7	I-166	Glycyrrhiza	glabra	Iran, Kerman	55°43′ 26.61″	29˚20′ 41.76″	1708
Taft-8, −9	I-167, I-168	Glycyrrhiza	glabra	Iran, Yazd	53°50′ 59.3″	31˚39′ 44.1″	2286
Takestan-2, −4	I-169, I-170	Glycyrrhiza	glabra	Iran, Qazvin	36˚06′ 59.43″	49°39′ 19.04″	1393
Yasuj-1, −10, −3, −5, −6	I-171 to I-175	Glycyrrhiza	glabra	Iran, Kohgiluyeh and Boyer-Ahmad	51°36′ 33.58"	30˚32′ 11.95"	2002

^*^ The names of the provinces and the geographical coordinates of the samples collected in the wild are taken from Esmaeili et al. [7, 15]

^**^ Botanical Garden of the University of Veterinary Medicine Vienna

In addition, we used 13 phenotypic traits in a panel of 66 *G. glabra* individuals collected from 22 regions in Iran (Table S2). These phenotypic and phytochemical trait data used for association analysis have been published in our previous studies [7, 15]. The phenotypic traits were fresh and dry weight, plant height and diameter, stem diameter, number of branches, leaf length and width. The average value of glabridin and liquiritigenin in the fleshy texture of root was 0.31 and 0.77 (mg/g DW) respectively, while the range of these metabolites in the cork layer of root was 2.62 and 0.53 (mg/g DW), respectively.

### Genotyping-by-sequencing and SNP detection

For the panel of genebank accessions, genomic DNA from fresh leaves was extracted with the DNeasy Plant mini kit (Qiagen, Hilden, Germany) following the manufacturer’s protocol. The DNA from the Iranian populations was isolated from fresh leaves with the CTAB procedure as described by Doyle and Doyle [26]. A Nanodrop spectrophotometer (A260/280, ND-1000 PEQLAB Biotechnologie GmbH) was used to verify the purity of the DNA, and an 1.3% agarose gel was used to control the integrity of the DNA. In total, 200 ng DNA per sample were digested with PstI and MspI (New England Biolabs) and processed for GBS library construction as described by Zhang et al. [27]. Equimolar pools of the individually barcoded samples (unique dual indexes) were sequenced (Illumina Novaseq 6000 device, SPflowcell, XP-workflow, single read, 107 cycles) using a custom sequencing primer [28] according to the manufacturer's instructions (Illumina Inc., San Diego, CA, United States).

After genotyping-by-sequencing (GBS) of 210 individuals, we removed the samples with low quality reads. At first, filtering was performed based on the mean of whole read counts for each sample by CLC Genomics Workbench software v.21. These individuals were preserved which included at least 1 million reads. Finally, 35 samples were removed and the remaining 175 samples were used for the subsequent analyses.

Trimming, mapping, clustering, and SNP calling were performed using the Linux-based software pipeline pyRAD (v.0.9.93) [29]. If not described differently, the default settings were used. The minimum number of samples per locus for SNPs calling was set to 80%. For the mapping of 175 GBS samples, we used the NCBI *Glycyrrhiza uralensis* chromosome level reference genome, cultivar manju (BioProject PRJNA787406, ID 787406; assembly number GCA_027886165.1_GRF_GUR_1.0; National Center for Biotechnology Information, U.S. National Library of Medicine, https://www.ncbi.nlm.nih.gov/bioproject/PRJNA787406. The parameter for the clustering threshold of reads within and between individuals was set to 0.85, and filtering for barcodes, adapters, and cut sites was done strictly by setting the value “1”. The maximum ploidy level was set as diploid. The pyRAD pipeline identified 38,393 SNP sites from 175 individuals according to these criteria.

### Analysis of the genetic diversity and genetic structure

The genetic diversity and structure of the *Glycyrrhiza* spp. panel was analyzed using the Jupyter package 7.2.2 [30] in pyRAD using the filtered 38,393 SNP markers from the pyRAD analysis. In addition, the optimal number of genetic clusters (K) was determined using the Bayesian clustering algorithm implemented in STRUCTURE v2.3.4 [31], with the most likely K identified based on the Evanno method calculated via the online platform Structure Harvester [31], and the results from multiple independent runs were aligned and visualized using CLUMPAK [32].

### Heterozygosity

We used VCFtools and Plink 2 to calculate the heterozygosity in the population and then plotted the results using ggplot 2 in R.

### Phylogenetic tree

We used the RAxML (Randomized Accelerated Maximum Likelihood) package in Linux to make a phylogenetic tree using a PHYLIP file containing the 38,393 SNP markers from the pyRAD analysis. Overall, a full ML analysis with 20 threads, GTRGAMMA model, and 500 bootstrap replicates was performed. As out-group(s) the other species than *G. glabra* were confirmed. To validate the results from the ML and STRUCTURE analysis, Principal Component Analysis (PCA) was done using the ggplot2 package in R.

### Analysis of phenotype-genotype associations

To obtain more reliable SNPs for association studies the SNP sites identified by the pyRAD pipeline were filtered using the VCFtools package in Linux. We applied the missing data = 10% and MAF ≥ 10% on the 38,393 SNPs and finally detected 14,651 SNPs. Imputation of missing data is strongly recommended in genetic association studies [33], as it limits the discovery of false positives. Therefore, to be sure about the quality of the SNPs, imputing was applied using Beagle software on 14,651 SNPs. Then the missing data was rechecked by TASSEL software and finally, 14,530 SNP markers were identified.

An association study was performed for 13 phenotypic traits (Table S2) in a panel of 66 individuals collected from different regions in Iran, based on 14,530 SNPs with a Mixed Linear Model (MLM) that incorporated three PCs and a kinship matrix using the R package GAPIT version 3.4 [34].

The significance threshold for SNP–trait associations was the Bonferroni correction of *P-value* ≤ 0.05/n, where n represents the number of SNP markers in the whole genome. For candidate gene analysis, genomic regions within the LD block of significant SNPs (peak SNPs) were selected to identify candidate genes. The DNA sequences containing highly significant markers as identified by the association study were annotated using BLAST (BLASTn, BLASTx, and tBLASTx) against the NCBI plant genomes nucleotide collection of flowering plants data using the dis-contiguous megaBLAST algorithm. Mapping and annotation of the BLAST results were performed using BLAST2GO and default settings. Additionally, BLASTn was performed against *Glycyrrhiza uralensis*. Local BLAST analysis was done with CLC Genomics Workbench.

## Results

### Genetic structure and phylogenetic tree reveal that all of the Iranian species are G. glabra

**Fig. 1:**
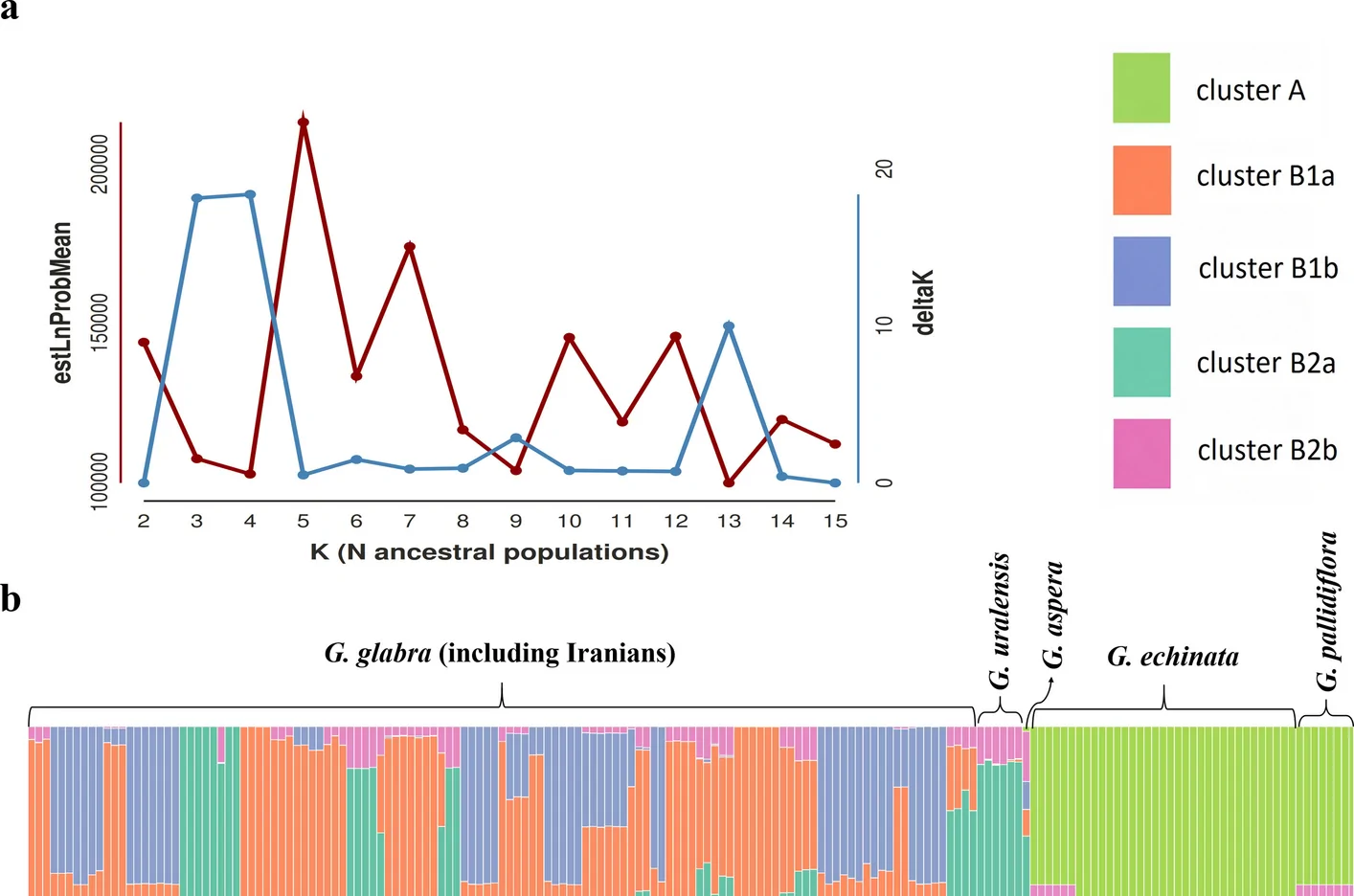
The genetic diversity and structure of *Glycyrrhiza* spp. **a** shows the parameter *K*, which describes the number of subpopulations in the total populations/accessions, and **b** shows the structure of the populations/accessions (STRUCTURE v2.3.4)

The genetic structure analysis of the population of 175 individuals using 38,393 SNPs confirmed that at least 5 different genetic clusters can be discriminated in our panel (Fig. 1 a, b). Among the Iranian samples of *G. glabra*, many were distinct from the samples of *G. glabra* from the genebank Gatersleben as these Iranian ones were dominated by the blue-colored genetic cluster, whereas others were genetically similar to the ones from the IPKs genebank. Overall, the panel of *G. glabra* populations/accessions displayed a high genetic diversity. The plants from *G. echinata* and *G. pallidiflora* were grouped together and genetically clearly distinct from the other plants (Fig. 1b and table S1: the samples dominated by the light green colored clustered at the right of the barplot). The one plant from *G. aspera* (GLY20-1) seemed to be distinct from the other samples, as it was the only one characterized by a mixture of all five clusters, which no other sample showed. As expected, the 4 different species of *G. uralensis*, *G. echinata*, *G. pallidiflora*, and *G. aspera* as outgroups were genetically distinguishable from *G. glabra* (Fig. 1). However, the genetic structure of some *G. glabra* plants from genebank accessions was similar to the G*. uralensis* plants (e.g. GLY11-6, GLY9-1, Gly9-2), i.e. dominated by the turquoise cluster with a minor portion of the rose cluster (Fig. 1, Table S1). This indicated a certain genetic relatedness between *G. glabra* and *G. uralensis*.

**Fig. 2:**
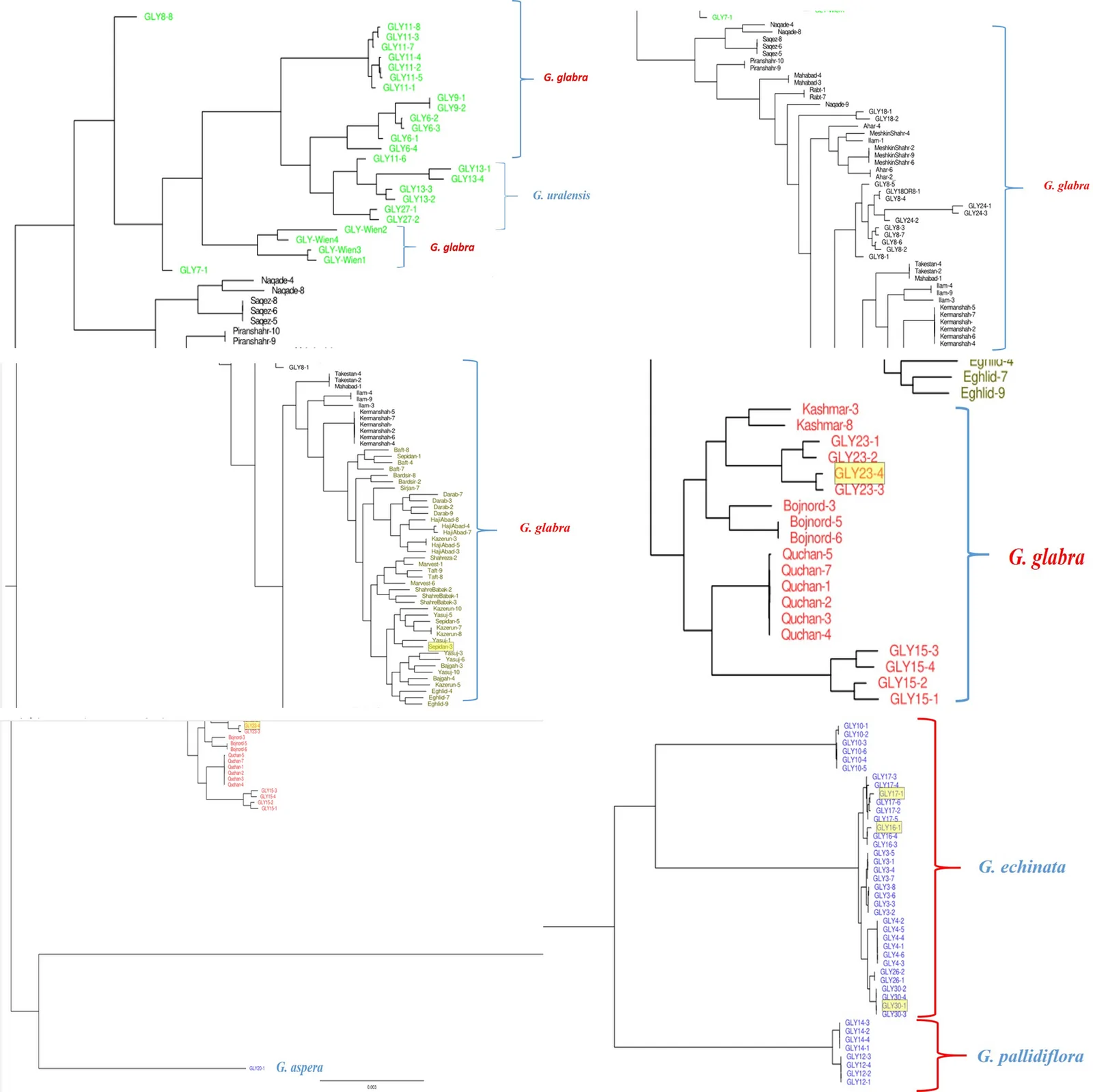
Phylogenetic tree of *Glycyrrhiza* spp. calculated with the Maximum Likelihood method (ML). RAxML Linux package was used to draw the phylogenetic tree using 38,393 SNPs for 175 individuals from five species. A full ML analysis with 20 threads, GTRGAMMA model, and 500 bootstrap replicates was performed. The tree was rooted with the outgroup *G. aspera*. As the tree is big, we have divided it to show the single branches separately. The original, unbroken figure with bootstrap values is presented in Fig. S1

As shown by an ML analysis (Fig. 2), *G. uralensis* and *G. glabra* were genetically rather similar. GLY 7 and VIENNA1/= GLY-Wien (*G. glabra*) were grouped close to the *G. uralensis* samples. The *G. echinata* and *G. pallidiflora* genotypes were grouped in a different branch but were more closely related to each other than to the other *Glycyrrhiza* species***.**** G. aspera* was classified in a completely separate branch (Fig. 2, Fig. S1).

**Fig. 3:**
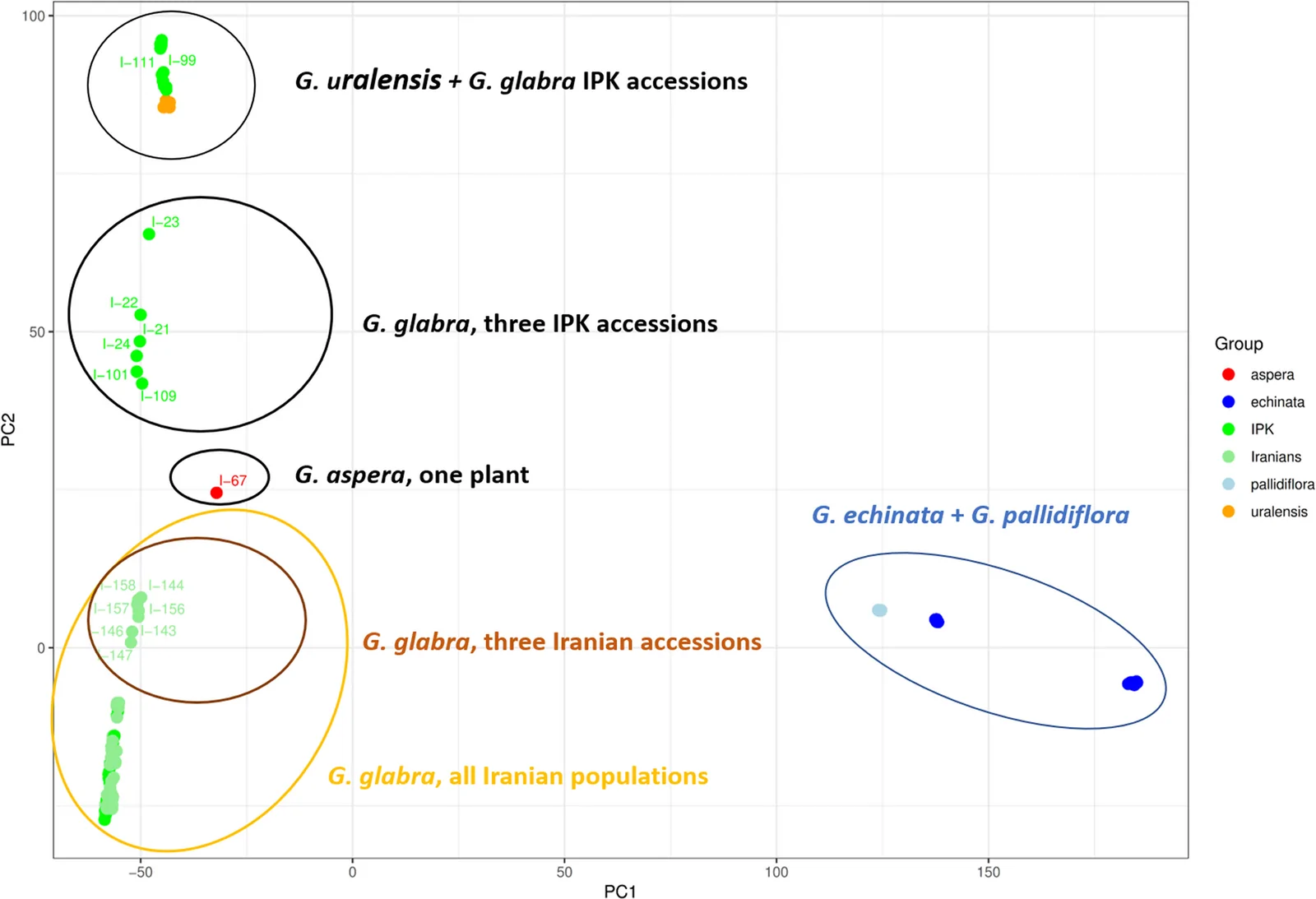
Principal Component Analysis (PCA) using 38,393 SNPs in 175 individuals based upon PC1 (81.1%) and PC2 (5.2%)

To validate the results from the phylogenetic tree and population structure analyses, PCA was done using the ggplot2 package in R. These individuals were divided into five main groups. As Fig. 3 shows, the results from the Principal Component Analysis (PCA) plot corresponded to both the STRUCTURE and phylogenetic tree analyses. The broad dispersion occurs along the first two principal components (Fig. 3). For instance, the 175 analyzed individuals were divided into five main groups, with the group of Iranian *G. glabra* samples displaying considerable genetic diversity while being clearly separated from the *G. uralensis* samples. Thus, these results were validated.

### Heterozygosity

**Fig. 4:**
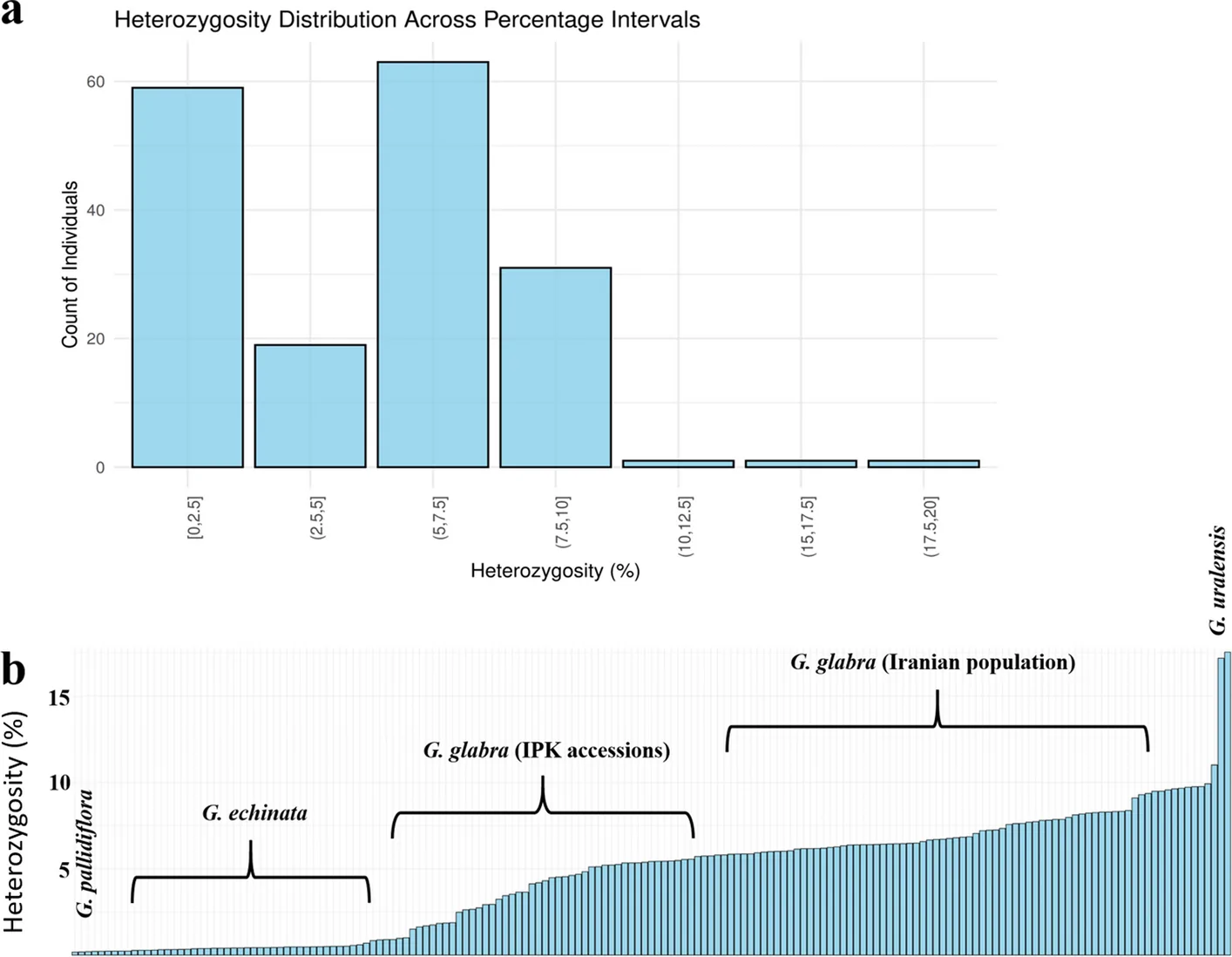
The heterozygosity in the population for the GBS data. **a** shows the heterozygosity percent in the population, and **b** shows the heterozygosity percent by individuals

As shown in Fig. 4, the heterozygosity for the individuals was for nearly all samples below 10%, with nearly 60 samples showing a heterozygosity value below 2.5% (Fig. 4a). The Iranian *G. glabra* individuals showed a higher heterozygosity (Fig. 4b), but the highest heterozygosity was identified in GLY8 (20%; *G. uralensis*) and GLY7 (20%; *G. glabra*), and the least heterozygosity was found in GLY12 and GLY14 (*G. pallidiflora*). 44 individuals showed a heterozygosity close to 0, i.e. < 0.5% (Fig. 4b), including all accessions of *G. echinata*.

### Association study for secondary metabolites in G. glabra

**Fig. 5:**
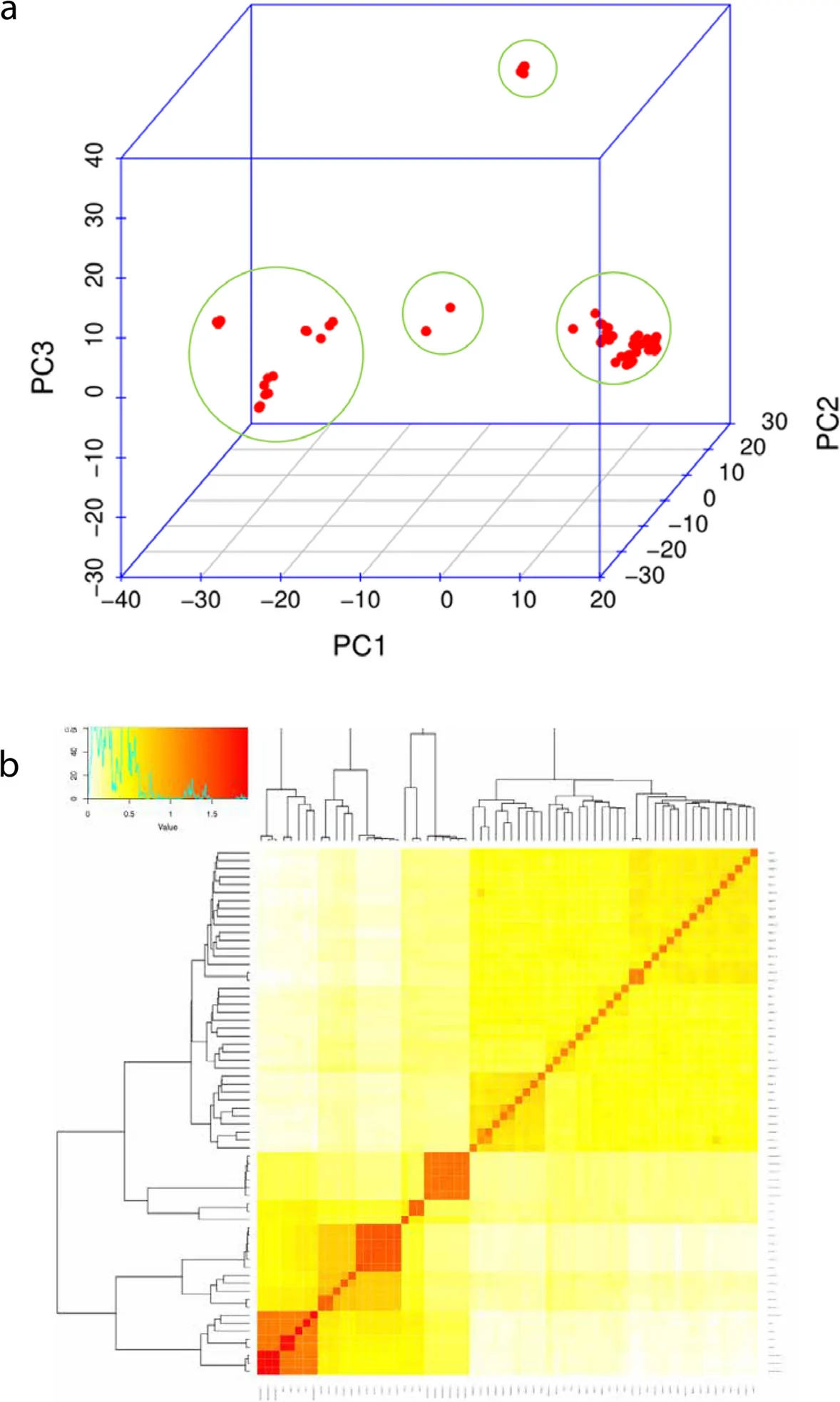
Genetic relationship for the 66 Iranian *G. glabra* individuals. a Four main groups could be identified by a 3D-PCA. **b** The kinship correlation matrix also grouped the individual plants into four main clusters

An association analysis was performed for 13 traits from 66 individuals collected in 22 regions of Iran, based on 14,530 SNPs with a Mixed Linear Model (MLM) that incorporated three PCs (Fig. 5a) and a kinship matrix (Fig. 5b). These individuals were divided into four main groups (Fig. 5a, Fig. 5b).

SNP-loci significantly associated with three traits were identified (Table 2). The association analysis resulted in the identification of 3 different SNPs for glabridin in the fleshy texture of the root, 15 SNPs for glabridin in the cork layer, and 5 SNPs for liquiritigenin in the cork layer of the root, all of which were above the defined significance threshold *P-value* 0.05 under Bonferroni correction (Table 2). The three SNPs that were associated with the glabridin content in the fleshy (parenchymatous) portion of the root were likewise associated with glabridin content in the root’s cork (periderm) layer.

**Table 2:** Association study identified SNPloci associated with traits. The significance threshold *P-value* under Bonferroni correction is listed

	**SNP**	**Chromosome**	**Position**	**P-value**		**Effect size**		**% Variance**
Glabridin in Fleshy texture of Root								
1	loc1777_pos197	CM051127.1	9,425,912	4.54E-07		1.25		10.5
2	loc2253_pos30	CM051127.1	39,156,166	2.29E-06		0.85		6.2
3	loc4345_pos47	CM051130.1	5,002,290	4.54E-07		1.18		9.8
Glabridin in Cork layer of Root								
1	loc270_pos248	CM051125.1	21,495,205	3.35E-06		0.78		5.1
2	loc892_pos25	CM051126.1	404,370	9.29E-08		1.45		12.3
3	loc1616_pos22	CM051126.1	41,944,749	4.07E-07		1.3		11
4	loc1777_pos197	CM051127.1	9,425,912	3.75E-07		1.22		10.2
5	loc1885_pos14	CM051127.1	18,620,703	3.22E-07		1.35		11.5
6	loc1886_pos25	CM051127.1	18,623,531	3.22E-07		1.33		11.3
7	loc1935_pos16	CM051127.1	23,631,646	4.07E-07		1.28		10.8
8	loc1935_pos28	CM051127.1	23,631,658	4.07E-07		1.26		10.6
9	loc1935_pos30	CM051127.1	23,631,660	4.07E-07		1.24		10.3
10	loc2253_pos30	CM051127.1	39,156,166	2.58E-06		0.82		5.8
11	loc3723_pos196	CM051129.1	2,106,807	9.29E-08		1.5		12.8
12	loc4345_pos47	CM051130.1	5,002,290	3.75E-07		1.2		10
13	loc4658_pos24	CM051131.1	12,268,767	9.29E-08		1.48		12.6
14	loc5330_pos33	CM051132.1	2,692,432	9.29E-08		1.42		12
15	loc5427_pos101	CM051132.1	6,467,438	3.39E-06		0.75		4.9
Liquiritigenin in Cork layer of Root								
1	loc468_pos9	CM051125.1	31,495,268	3.11E-06		0.8		5.5
2	loc634_pos100	CM051125.1	36,094,394	8.36E-07		1.1		9.2
3	loc842_pos32	CM051125.1	43,154,203	8.36E-07		1.05		8.8
4	loc1849_pos3	CM051127.1	15,200,011	8.36E-07		1.08		9
5	loc2166_pos64	CM051127.1	35,905,736	3.89E-07		1.15		9.5

The BLAST (Basic Local Alignment Search Tool) of the sequences harboring significantly associated SNPs for glabridin and liquiritigenin against the NCBI nucleotide collection of flowering plants identified significant hits to potential candidate genes (BLAST-score of > = 80 with a sequence identity of > = 80% and an E-value < 1 × 10 − 13) in multiple plant species.

### The sequences harboring significantly associated SNPs for glabridin in the fleshy texture of the root

**Fig. 6:**
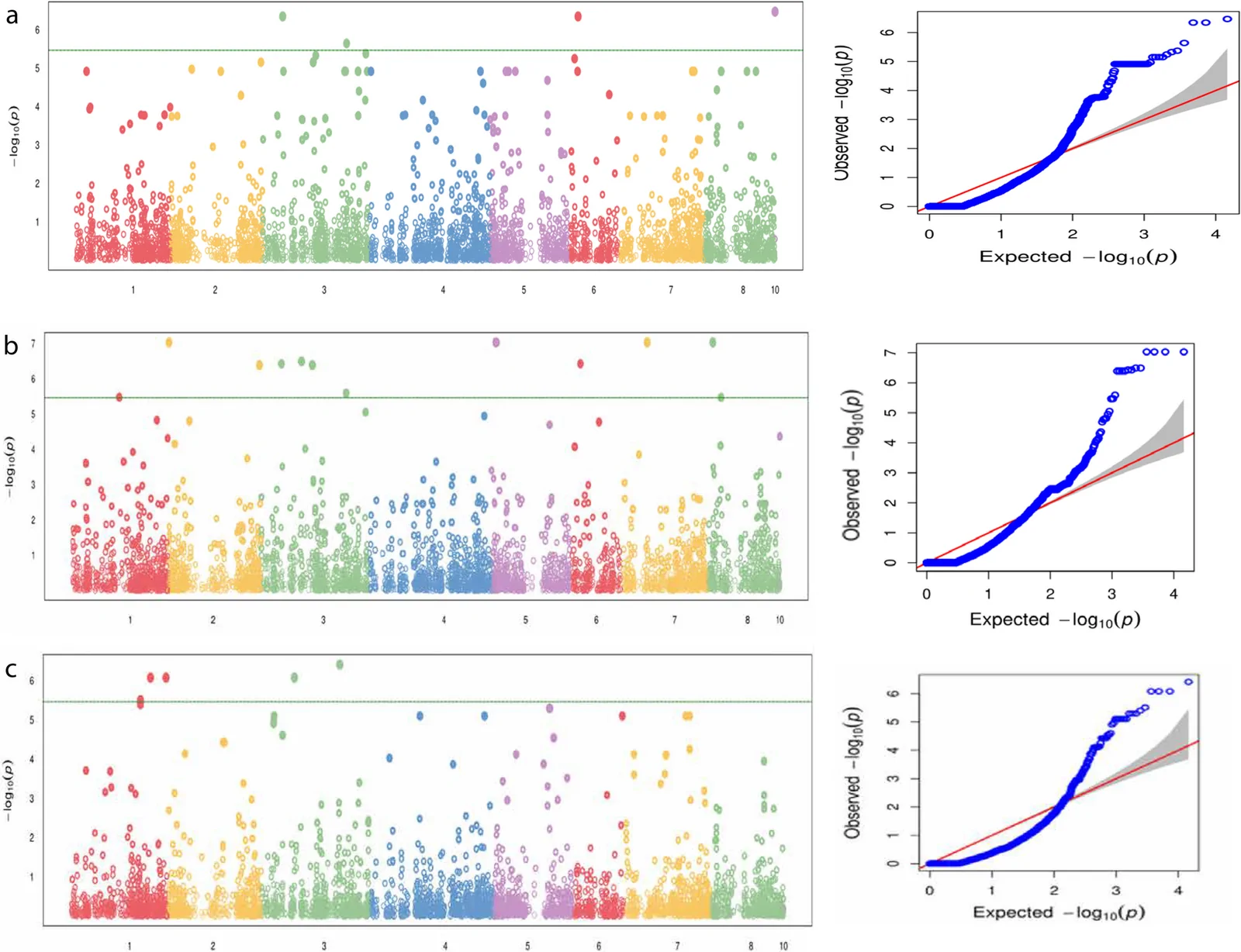
Association study results for secondary metabolites in the root of *G. glabra* using MLM: Left: Manhattan plots. Each dot represents one SNP. The numbers on the horizontal axis represent the chromosome numbers. The Bonferroni threshold of − log10(P) = 5.5 is represented by a green line on the Manhattan plots. The significantly associated SNPs were located above the green line. Right: Quantile–quantile (Q–Q) plots. The plot shows the expected versus observed − log10(P) of each marker (blue dots). The red line is a benchmark for the perfect fit to the expected − log10(P). The gray shaded area shows the 95% confidence interval for the Q–Q plot under the null hypothesis of no association between the SNP and the trait. **a** Association study results for the trait glabridin in the fleshy texture of the root. **b** Association study results for the trait glabridin in the cork layer of the root. **c.** Association study results for the trait liquiritigenin in the cork layer of the root

Manhattan Quantile–quantile (Q–Q) plots clearly illustrated some significant SNPs (Fig. 6a). BLAST analysis of the sequences harboring significantly associated SNPs for glabridin identified one genomic region. We identified transcription repressor MYB6 (XP_019451270.1), E3 ubiquitin-protein ligase UPL1 (XP_014630062.1), and protein OCTOPUS-like (XP_006573340.1).

### The sequences harboring significantly associated SNPs for glabridin in the cork layer of the root

Manhattan Quantile–quantile (Q–Q) plots clearly illustrate several significant SNPs (Fig. 6b). The BLAST analysis of the sequences harboring significantly associated SNPs for glabridin identified the following genomic regions: Mitochondria cytochrome c oxidase subunit 1 (YP_008999560.1), Transcription factor bZIP63 (ABP88245), F-box/LRR-repeat protein 14 (Q5VMP0), Transcription factor MYB41 (KAH1230721.1) or transcription repressor MYB6-like (XP_019451270.1), Chloroplastic protein DETOXIFICATION 44 (Q945F0), Protein NRT1/PTR FAMILY 8.1 (XP_003551296.1), cell division control protein 48 homolog C (XP_003531589.1), 3-ketoacyl-CoA synthase 11 (XP_003536489.1), E3 ubiquitin-protein ligase UPL1-like (XP_003527888.3), WRKY transcription factor 22 (XP_006591525.1), Protein OCTOPUS-like (XP_028224754.1), Dynamin-related protein 1E (XP_003532226.2), CCCH-type zinc finger family protein with RNA-binding domain (XP_003517520.1), and Putative RING-H2 finger protein ATL21A (RZC01568.1).

### The sequences harboring significantly associated SNPs for liquiritigenin in the cork layer of the root

Manhattan Quantile–quantile (Q–Q) plots clearly illustrate several significant SNPs (Fig. 6c). The BLAST analysis of five sequences harboring significantly associated SNPs for liquiritigenin in the cork layer of root identified several genes like dentin sialophosphoprotein isoform X4 (XP_027365055.1), Aminodeoxychorismate synthase, chloroplastic-like (XP_028219431.1), Spliceosome-associated protein 130 A (XP_003556541.1), 7-deoxyloganetin glucosyltransferase (XP_003545681.1), and Protein LAZ1 homolog 1 isoform X1 (XP_003527968.1) related to these traits (Table 2).

## Discussion

Assessing genetic diversity in plants is crucial, as it enables effective management and breeding, supports conservation initiatives, and facilitates the utilization of valuable germplasm [15, 35]. Genetic variation among and within populations is influenced by biological traits, distribution patterns, life history, population density, gene flow, mutation frequency, genetic drift, and mating systems [36, 37]. However, the genetic differentiation among populations is described to be largely dependent on the mating system, i.e. inbreeding vs. selfing, with selfing leading to reduced gene flow and higher genetic drift [38]. For licorice especially with focus on *G. glabra*, this might be reflected in the genetic diversity observed across its populations in this study. Licorice are perennial, mostly autogamous herbs. While *G. glabra* is under quarantine in many regions of Iran due to excessive harvesting, this research has shown that it possesses a high level of diversity, potentially reflecting its ability to adapt to different environmental conditions. Thus, studies on population genetics of different *Glycyrrhiza* spp. are crucial to provide the foundation for understanding the evolutionary history and adaptive potential of these medicinally and economically important plants. For instance, there is a significant genetic differentiation among core species like *G. glabra*, *G. uralensis*, and *G. inflata* at the whole-genome level described [19], reflecting long-term geographic and ecological isolation that has reduced gene flow [11].

The genetic diversity of *Glycyrrhiza spec.* including *G. glabra* has been explored in several studies using AFLP [39] and ISSR (inter-simple sequence repeat) and RAPD (random amplification of polymorphic DNA) [40–42]. ISSR and RAPD revealed that 81–85% of total genetic variation resided within populations, with moderate genetic differentiation, suggesting shared ancestry with regional divergence, and STRUCTURE analysis identified three major genetic clusters [17]. While these markers successfully revealed significant genetic diversity, they suffer from low marker density, an inability to identify specific trait-associated loci, and a restricted reproducibility [43]. By an AFLP study, three markers significantly associated with glycyrrhizin concentration and four markers linked to phenolic compound content were identified [13]. However, AFLP markers are dominant and have limited transferability and genome coverage, and thus have a restricted use for practical plant breeding. A study on Iranian *G. glabra* populations using SSR markers, in combination with morphological and phytochemical analyses, revealed significant population structure and genetic variation [15]. Even though SSR markers offer advantages in terms of reproducibility and codominance, they still provide limited genome coverage compared to high-throughput approaches as well as whole-genome and phylogenomic approaches.

Advanced sequencing methods such as genotyping-by-sequencing (GBS) enable to perform a much more extensive evaluation of genetic diversity, resulting in thousands of high-quality markers that can be used to assess genetic variation in plants [e.g. 22, 44]. In this study, we analyzed the genetic diversity of 175 *Glycyrrhiza* spp. individuals using 38,393 SNPs resulting in the identification of 5 main groups, with the group of Iranian *G. glabra* samples displaying considerable genetic diversity (Fig. 1 and 3). The species *G. echinata* and *G. pallidiflora* were grouped together and genetically clearly distinct from the other individuals (Fig. 1b), as also indicated by a bootstrap support of 100% in the phylogenetic reconstruction (Fig. S1). The one sample from *G. aspera* appeared to be distinct from the other samples (Fig. 2 and Fig. 3), indicating its strong genetic difference to the other *Glycyrrhiza* species. As expected, the 4 different species of *G. uralensis*, *G. echinata*, *G. pallidiflora*, and *G. aspera* as outgroups were largely separated from *G. glabra* (Fig. 2). However, *G. uralensis* and *G. glabra* were genetically rather similar though: Some *G. glabra* accessions are grouped close to the *G. uralensis* samples (Figs. 1b, 2, and 3). Viable and fertile hybrids between *G. glabra* and *G. uralensis* are reported to occur frequently and were partly even classified as own species, i.e. *G. korshinskyi* [11], confirming the applicability of a *G. uralensis* reference genome to research in *G. glabra*. A whole-genome re-sequencing study state *G. glabra* and *G. inflata* as closely related, while both showed higher differentiation from *G. uralensis* [19].

Some studies have evaluated licorice diversity using different molecular markers. For example, Esmaeili et al. [15] have subdivided 162 Iranian *G. glabra* populations into 5 groups using SSR markers. Our SNP analysis enabled us to identify genetic clusters within *Glycyrrhiza* spp., with some genotypes predominantly belonging to a single genetic group, while others are consisting of different genetic groups (Fig. 1b), potentially an evidence of introgression and crossings. Some origins have been identified as genetically distinct from most other origins, which could be a source for breeding to introgress interesting genetic material in novel, superior varieties of *G. glabra* and *G. uralensis*. High genetic differentiation (Fig. 2) and low heterozygosity (Fig. 4) indicate a general long-term geographic and ecological isolation resulting in reduced gene flow, as also described by Kim et al. [11]. An average low heterozygosity below 0.01 in *Glycyrrhiza* species is confirmed by Kim et al. [11] with the lowest one described for *G. pallidiflora* with approx. 0.001, a range consistent with our results (Fig. 4).

GWAS using high-density SNP markers across well-phenotyped populations remain underutilized for licorice. Such studies exploit historical recombination, enabling mapping to narrower genomic intervals than e.g. AFLP-based association mapping. As example, the use of GWAS allows to identify stress-tolerance genes by utilizing genetically diverse populations [44]. The chromosome-level reference genomes for *G. uralensis* provide a robust foundation [11]. Association mapping identified some key genes significantly associated with three main secondary metabolites in our study. As following steps, functional validation would be an important step to unravel the underlying genetics, as also the generation and analysis of segregating biparental or multiparental populations for the traits glabridin and liquiritigenin could help to dissect their genetics. As further following steps, the GWAS results can be combined with whole-genome resequencing, transcriptomics including transcription factors analysis [45], and metabolomics to confirm candidate genes and significantly improve resolution and statistical power [19]. The candidate genes found could be further examined for superior genotypes, which might direct efforts to improve crops through genome editing [44].

We conducted an association study for 13 secondary metabolites to identify marker trait associations (MTA). The power of a GWAS is determined by several factors, of which the sample size is one. With 66 samples, we used a small population for a GWAS. However, also small populations can successfully yield meaningful results in GWAS studies, e.g.: Hoyos-Villegas et al. [46] (96 common bean genotypes), Otto et al. [47] (91 *Matricaria recutita* plants, i.e. German chamomile), or Atwell et al. [48] (107 *A. thaliana* phenotypes). QTLs can also be detected in smaller populations if they explain a large proportion of the genetic variation. Based upon our phenotyping and genotyping data, we applied strict criteria for our analysis, and yielded for three of the 13 traits significantly associated SNP-marker, which is an important resource to improve licorice through breeding and to build a good basis for further investigations. It can be expected, that with a larger panel a GWAS would yield further MTAs, which we did not detect in our study.

Our GWAS results provide genetic evidence for the involvement of specific transcription factors and signaling proteins in glabridin accumulation [49]. The significantly associated SNPs for glabridin in the fleshy texture of the root identified three genes. Significantly associated SNP for glabridin content in the fleshy root tissue that mapped to a gene encoding the transcription repressor MYB6 (XP_019451270.1) were identified. MYB6 (XP_019451270.1) is a helix-turn-helix transcription factor known to upregulate anthocyanin and proanthocyanin biosynthesis while repressing lignin metabolism through interaction with the KNAT7 transcriptional repressor [45].. The association of MYB6 with glabridin, a prenylated flavonoid, suggests that this transcription factor may regulate multiple branches of the phenylpropanoid pathway, similar to its role in Arabidopsis MYB75 [50]. Given that MYB co-expresses with several stress-responsive transcription factors (bHLH, GRAS, NAC, WRKY) in *Glycyrrhiza* species [18], this gene may represent a key regulatory hub linking abiotic stress responses to secondary metabolite production. Our results confirm the findings of Liu et al. [45], who demonstrated that MYB6 represses lignin metabolism in poplar. However, we extend this observation to show that this gene is also associated with glabridin accumulation in licorice roots.

In addition, E3 ubiquitin-protein ligase UPL1 (XP_014630062.1) has shown significant alignments to many plant species. Ub-ligase proteins are established regulators of defense-related signaling component accumulation [51], with E3 ligases acting as key modulators of jasmonic acid (JA) and salicylic acid (SA) hormonal pathways that orchestrate coordinated responses [52]. One study confirmed that treatment with methyl jasmonate (MeJA) can significantly increase the accumulation of glabridin in licorice hairy root cultures [53]. Another study directly compared the effect of MeJA and SA on flavonoid production in *G. glabra* hairy roots and found that MeJA was effective at promoting flavonoid accumulation, while SA was less effective in promoting these effects [54].

The third identified gene was the protein OCTOPUS-like (XP_006573340.1) that potentiates primary root protophloem differentiation and regulates root architecture [55]. OCTOPUS is a positive regulator of the brassinosteroid signaling pathway [56]. In contrast to the findings of Wang et al. [57], our study suggests that the OCTOPUS may have a broader role in root architecture regulation beyond its known function in protophloem differentiation.

The significantly associated SNPs for glabridin in the cork layer of the root identified several important genes. We identified mitochondria cytochrome C oxidase subunit 1 (COX, YP_008999560.1). Expression of nuclear genes coding for COX proteins is particularly connected to hormonal control of plant growth. Plant hormones are connected to mitochondrial activity through abscisic acid (ABA), auxins, cytokinins (CKs), jasmonic acid (JA), and salicylic acid (SA), regulators of the expression of nuclear genes encoding mitochondrial components [58]. Several genes for COX structural components or editing and assembly factors are preferentially expressed in roots and endoreduplicative giant cells, in the shoot apical meristem, and at different stages during embryogenesis [59]. COX components are preferentially expressed in plant tissues with high energy demands, like shoot and root meristems, anthers, and, significantly, during all stages of embryogenesis [59].

In addition, the transcription factor bZIP63 (ABP88245, basic region/leucine zipper motif) was confirmed. The bZIPs are known to regulate the biosynthesis of flavonoids and other secondary metabolites in various plant species [60–62]. Since glabridin is a specialized flavonoid, it falls within a class of compounds known to be regulated by bZIPs [60].

Further identified was F-box/LRR-repeat protein 14 (Q5VMP0), which is involved in strigolactone (SL) signaling [63]. Strigolactones (SLs) are a class of diverse phytohormones derived from the carotenoid pathway [64]. F-box/LRR-repeat protein controls tillering by suppressing axillary bud activity [65]. A tiller is a specialized grain-bearing branch that is formed on the unelongated basal internode and grows independently of the mother stem (culm) using its own adventitious roots [65]. They function as crucial signaling components that regulate various aspects of root architecture. Some LRR proteins are part of E3 ubiquitin ligase complexes that regulate protein degradation in signaling cascades [66].

Concerning the transcription factor MYB41 (KAH1230721.1) or transcription repressor MYB6-like (XP_019451270.1) it is particularly interesting that this protein was also implicated in glabridin accumulation in the fleshy root texture, suggesting we have identified two isoforms of this protein involved in glabridin metabolism in root tissues.

Chloroplastic protein DETOXIFICATION 44 (Q945F0) functions as a multidrug and toxin extrusion transporter in the export of salicylic acid (SA) from the chloroplast to the cytoplasm [67]. It plays an essential function in plant defense via pathogen-induced salicylic acid (SA) accumulation and acts as a key component of the age-related resistance (ARR) pathway [68]. These transporters are known to mediate the transport and accumulation of various plant secondary metabolites, including those from the flavonoid family to which glabridin belongs [69].

A very interesting gene is NRT1/PTR FAMILY 8.1 (XP_003551296.1), a peptide transporter that also transports the plant hormones auxin (indole-3-acetic acid), abscisic acid (ABA), and gibberellin (GA), as well as secondary metabolites (glucosinolates) [70]. Crucially, NPF transporters are known to transport flavonoids, including the specific flavonol diglucosides found on pollen coats. This demonstrates that the NPF family has the capacity to handle molecules similar to glabridin [71].

Three SNPs corresponded to *CELL DIVISION CONTROL PROTEIN48* (CDC48, XP_003531589.1) and *3-ketoacyl-CoA synthase 11* (XP_003536489.1). CDC48 is essential for cell division (cytokinesis) and is highly expressed in rapidly growing tissues like shoot and root tips [72]. It interacts with certain N-ethylmaleimide-sensitive factor attachment protein receptors as part of specialized membrane fusion events where vesicles from the same organelle fuse; vesicle trafficking is a fundamental mechanism for maintaining cellular homeostasis in eukaryotes [73]. The 3-ketoacyl-CoA synthase 11 contributes to cuticular wax and suberin biosynthesis being involved in both decarbonylation and acyl-reduction wax synthesis pathways [74].

Another isoform of E3 ubiquitin-protein ligase UPL1-like (XP_003527888.3) is in conjunction with UPL1 in the fleshy root. This finding suggests that we have identified two isoforms of this protein that are involved in the glabridin metabolism in the root [53].

Another significant association for glabridin in the cork layer mapped to WRKY transcription factor 22 (XP_006591525.1). WRKY TFs are well known for regulating plant abiotic and biotic stress tolerance. WRKY22 likely regulates glabridin biosynthesis by modulating the expression of phenylpropanoid pathway genes, a mechanism observed in other plant species where WRKYs regulate the production of alkaloids, terpenes, and phenylpropanoids [75]. This finding expands our understanding of WRKY transcription factors beyond stress responses to include regulation of commercially valuable secondary metabolites. The role of WRKYs in the development, including trichome and root hair formation, seed coat color, seed size, plant senescence, and male gametogenesis, is also well documented [73].

Protein OCTOPUS-like (XP_028224754.1), as previously mentioned, was also implicated in glabridin accumulation in the fleshy root texture, further confirming that we have identified two isoforms of this protein involved in glabridin metabolism of the root.

The next significant association for glabridin in the cork layer was mapped to dynamin-related protein 1E (DRP1E, XP_003532226.2). The role of DRP1E in vesicle-mediated transport is essential for delivering materials like the cellulose synthase complexes to the cell surface, a process crucial for normal root growth [74]. Three significant associations for glabridin in the cork layer included a CCCH-type zinc finger family protein with RNA-binding domain (XP_003517520.1), RNA-binding protein Y14 (XP_003517521.1). and hexokinase-3 (XP_003517518.1). The CCCH-type zinc finger family and RNA-binding protein Y14 regulate lignin and secondary cell wall biosynthesis, which are crucial for root structural development [75] and regulate other secondary metabolites like proanthocyanidins and ginsenosides [76]. The hexokinase-3 (XP_003517518.1) is involved in root hair cell development by mediating cross-talk between glucose and ethylene response pathways [77]. It is expressed in roots, emerging lateral roots, and root meristems. This suggests that HXK3 helps coordinate sugar signaling with hormonal signals to regulate root growth and architecture [78].

A further significant association for glabridin in the cork layer was mapped to the putative RING-H2 finger protein ATL21A (RZC01568.1) that functions as E3 ubiquitin ligases and participates in plant drought tolerance via regulating ABA signal transduction [65]. The gene co-expression network analysis identified *ATL54* as highly co-expressed with genes for cellulose, lignin, and xylan biosynthesis [79]. In rice, the RING-H2 protein EL5 plays a crucial role in the maintenance of cell viability after the initiation of root primordia. It acts as a membrane-anchored E3 ligase that is rapidly induced by an elicitor and appears to be responsible for degrading cytotoxic proteins produced in root cells after hormonal signals [79].

The sequences harboring significantly associated SNPs for liquiritigenin in the cork layer of the root identified related important genes: phosphoprotein (XP_027365055.1), which may possess nucleic acid-binding activity, though its role in plants remains unclear. Phosphoproteomics revealed that phosphoproteins are involved in the MAPK signal transduction and brassinosteroid signaling pathways, which may lead to the accumulation of alkaloids and flavonoids [80]. Future phosphoproteomic studies on licorice could potentially identify regulatory phosphoproteins that control the liquiritigenin pathway. Aminodeoxychorismate synthase (ADCS), chloroplastic-like (XP_028219431.1) is another gene with a significant association for liquiritigenin in the cork layer. The central function of ADCS is to catalyze the first committed step in the synthesis of *para*-aminobenzoic acid (pABA), a precursor for folate (vitamin B9) [81]. Folate biosynthesis and metabolism are directly linked to proper root development. For instance, a mutation in the plastidial isoform of folylpolyglutamate synthetase (an enzyme downstream of ADCS in the folate pathway) was found to be required for postembryonic root development. Furthermore, research indicates that folate shapes plant root architecture by affecting auxin distribution [82].

Spliceosome-associated protein 130 A (XP_003556541.1) is a subunit of the splicing factor SF3B and required for 'A' complex assembly. Its primary function is to fine-tune JA signaling through alternative splicing [83]. Although it is not directly involved in liquiritigenin synthesis, JA is a key signaling molecule that promotes the accumulation of flavonoids in licorice, including liquiritigenin [84]. Another gene is 7-deoxyloganetin glucosyltransferase (XP_003545681.1) that is involved in various functions, including the regulation of hormone homeostasis, the detoxification of xenobiotics, and the biosynthesis and storage of secondary compounds controlled by a specific subclass of the ubiquitous glycosyltransferase family [85]. The last identified gene LAZ1 homolog 1 isoform X1 (XP_003527968.1) functions as a transmembrane organic solute transporter. The LAZY1 (LAZ1) protein family is well-characterized for its role in root development [86]. These LAZY proteins are key regulators of the gravitropic response, which determines the angle at which roots grow downward [86].

Our GWAS identified SNPs that can be deployed for breeding licorice genotypes with enhanced yields of glabridin, liquiritigenin, and glycyrrhizin, each linked to distinct precursor pathways. For liquiritigenin—the central flavanone precursor at the branch-point of the phenylpropanoid pathway—SNPs associated with aminodeoxychorismate synthase and 7-deoxyloganetin glucosyltransferase provide targets to modulate its accumulation, thereby controlling the precursor pool that feeds into both glabridin and other bioactive flavonoids such as liquiritin. For glabridin itself, tissue-specific SNPs associated with MYB6/MYB41, OCTOPUS-like and UPL1 could serve as markers to selectively enhance the downstream conversion of liquiritigenin into this high-value isoflavonoid in root tissues. For glycyrrhizin, a triterpenoid saponin derived from the independent mevalonate pathway via 2,3-oxidosqualene, SNPs in stress-responsive TFs (bHLH, GRAS, WRKY) provide levers to upregulate its biosynthetic cassette without competing for phenylpropanoid precursors. Genes involved in glycyrrhizin biosynthesis are conserved across the three *Glycyrrhiza* species *G. uralensis, G. inflata*, and *G. glabra* [3], and thus single markers might be usable for all three species.

Additionally, SNPs associated with root architecture genes (OCTOPUS-like, NPF8.1) and strigolactone signaling (F-box/LRR-repeat protein 14) enable selection for increased root biomass, expanding the sink capacity for all three metabolite classes. In future studies, further (experimental) validation of these identified markers in the frame of a functional analysis to proof the marker to gene to metabolite relationship would be beneficial, though.

Based on these identified genomic resources, genome editing technologies, particularly CRISPR/Cas systems, might provide a platform for accelerating breeding programs through sequence-specific and multi-site modifications [87]. These tools can be utilized in haploid breeding and de novo domestication, enabling the rapid introgression of superior alleles into elite backgrounds, as for instance described for salinity tolerance [88]. Collectively, the markers identified in this study contribute to a toolkit for marker-assisted selection, genomic selection and genome editing to develop elite *Glycyrrhiza* varieties with superior profiles of liquiritigenin, glabridin, and glycyrrhizin through the coordinated manipulation of their interconnected and independent precursor pathways.

## Conclusions

This study provides a foundational genomic resource for the medicinal genus *Glycyrrhiza* by employing genotyping-by-sequencing (GBS) to unravel its genetic architecture and link genetic variation to key medicinal compounds. Our analysis of 175 accessions using 38,393 high-quality SNPs revealed a clear genetic structure, successfully differentiating *G. glabra* from related species. Notably, the Iranian *G. glabra* populations formed a distinct, diverse cluster, underscoring their unique genetic identity and highlighting Iran as a significant reservoir of genetic diversity for this species. The core innovation of this work lies in the first genome-wide association study (GWAS) conducted for secondary metabolites in *G. glabra*. We identified 20 highly significant SNP markers associated with the accumulation of glabridin and liquiritigenin—two flavonoids with documented roles in plant stress response and human health—in the root's cork and fleshy layers. The co-localization of three SNPs for glabridin in both root tissues suggests a shared genetic regulatory mechanism for its biosynthesis or transport. Furthermore, the annotation of genomic regions flanking these significant SNPs pinpointed several compelling candidate genes. These include transcription factors like MYB6 and WRKY22, which are known master regulators of flavonoid and phenylpropanoid pathways; OCTOPUS-like, a regulator of root development and brassinosteroid signaling; and Aminodeoxychorismate synthase, a key enzyme in the folate biosynthesis pathway. The identification of these genes provided crucial initial insights into the genetic control of valuable medicinal compounds in licorice. In summary, our findings deliver a dual resource: a detailed map of genetic diversity within *Glycyrrhiza* spp. and a set of robust, trait-associated genetic markers. The identified SNPs and candidate genes established a critical platform for future marker-assisted selection, empowering the development of superior *Glycyrrhiza* cultivars with enhanced and reliable concentrations of high-value phytochemicals.

## Supplementary Information

Supplementary Material 1: Table S1. Population structure of our *Glycyrrhiza*spec. panel analysed by STRUCTURE software showing the detailed results for each single plant.

Supplementary Material 2: Table S2. Phenotypic values of the traits scored for the Iranian plant panel of *Glycyrrhiza* *glabra*.

Supplementary Material 3: Fig. S1. Unbroken phylogenetic tree of *Glycyrrhiza *spp. calculated with the Maximum Likelihood method (ML). RAxML Linux package was used to draw the phylogenetic tree using 38393 SNPs for 175 individuals from five species. A full ML analysis with 20 threads, GTRGAMMA model, and 500 bootstrap replicates was performed. The tree was rooted with the outgroup *G. aspera*.

## Data Availability

The raw read data for the examined populations and accessions of this study have been deposited in the European Nucleotide Archive (ENA) at EMBL-EBI under accession number PRJEB105592 (https://www.ebi.ac.uk/ena/browser/view/prjeb105592 */)*. All other data generated or analysed are included in this manuscript or in its supplementary information files.

## Abbreviations

ABA: Abscisic acid
AFLP: Amplified fragment length polymorphism
ARR: Age-related resistance
BLAST: Basic local alignment search tool
GA: Gibberellin
GBS: Genotyping-by-sequencing
GWAS: Genome-wide association study
GWAS: Genome-wide association study
ISSR: Inter-simple sequence repeat
MAF: Minor allele frequency
ML: Maximum likelihood method
MLM: Mixed linear model
NGS: Next-generation sequencing
PCA: Principal component analysis
RAPD: Random amplification of polymorphic DNA
RAxML: Randomized accelerated maximum likelihood
RFLP: Restriction fragment length polymorphism
SA: Salicylic acid
SNP: Single nucleotide polymorphism
SSR: Simple sequence repeat
