# Supplementary material for: Genetic structure analysis of Glycyrrhiza species and identification of SNP-loci associated with secondary metabolites in G. glabra
Source: BMC Genomics. 2026 Sep 18;27:767. doi: 10.1186/s12864-026-13361-y (PMC13589345; doi:10.1186/s12864-026-13361-y)
Supplement: Supplementary file 1 [file 12864_2026_13361_MOESM2_ESM.docx]

| **Population** | Fresh weight (g) | Dry weight (g) | Plant height (cm) | Plant diameter (cm) | Stem diameter (mm) | No. of branches | No. of node | Leaf length (mm) | Leaf width (mm) | Glabridin- FTR  (mg/g DW) | Glabridin- CLR  (mg/g DW) | Liquritigenin- FTR  (mg/g DW) | Liquritigenin- CLR  (mg/g DW) |
| --- | --- | --- | --- | --- | --- | --- | --- | --- | --- | --- | --- | --- | --- |
| Bajgah 1 | 68.80 | 33.82 | 34.77 | 22.73 | 5.70 | 11.65 | 9.29 | 33.21 | 8.92 | 0 | 0.23 | 0 | 0.69 |
| Bajgah 2 | 62.20 | 25.61 | 50.79 | 20.50 | 5.01 | 12.98 | 9.96 | 27.56 | 9.91 | 0 | 0.19 | 0 | 0.68 |
| Bajgah 3 | 70.70 | 29.76 | 40.44 | 20.72 | 5.22 | 14.02 | 8.63 | 29.69 | 10.48 | 0 | 0.2 | 0 | 0.67 |
| Meshkin shahr 1 | 50.10 | 22.58 | 30.26 | 12.85 | 5.78 | 3.06 | 10.50 | 36.37 | 10.58 | 0 | 7.73 | 0.32 | 0 |
| Meshkin shahr 2 | 46.80 | 16.56 | 26.11 | 12.75 | 5.63 | 9.53 | 9.54 | 24.42 | 11.95 | 0 | 7.69 | 0.3 | 0 |
| Meshkin shahr 3 | 43.30 | 19.38 | 22.64 | 12.66 | 5.13 | 9.89 | 8.12 | 32.48 | 13.06 | 0 | 7.85 | 0.32 | 0 |
| Marvest 1 | 55.85 | 20.82 | 31.71 | 18.64 | 5.22 | 9.86 | 6.95 | 31.03 | 13.03 | 0 | 1.11 | 0.69 | 0.74 |
| Marvest 2 | 34.26 | 15.61 | 27.68 | 17.64 | 4.93 | 12.36 | 10.13 | 36.48 | 12.74 | 0 | 1.01 | 0.7 | 0.75 |
| Marvest 3 | 42.41 | 18.07 | 30.80 | 19.05 | 5.09 | 12.28 | 7.92 | 35.99 | 11.50 | 0 | 1.02 | 0.67 | 0.73 |
| Kermanshah 1 | 51.07 | 22.13 | 27.12 | 13.35 | 4.61 | 11.65 | 8.50 | 24.96 | 8.47 | 0.65 | 6.81 | 0 | 0 |
| Kermanshah 2 | 31.16 | 17.44 | 29.73 | 13.45 | 4.05 | 9.20 | 7.64 | 30.11 | 10.23 | 0.7 | 6.73 | 0 | 0 |
| Kermanshah 3 | 35.27 | 19.36 | 33.09 | 13.80 | 4.12 | 10.43 | 6.32 | 29.63 | 11.16 | 0.62 | 6.66 | 0 | 0 |
| Kashmar 1 | 55.55 | 27.28 | 36.36 | 14.12 | 4.22 | 9.97 | 5.40 | 27.50 | 8.63 | 2.52 | 10.29 | 0.78 | 0 |
| Kashmar 2 | 39.85 | 21.22 | 28.68 | 14.23 | 4.63 | 12.13 | 6.74 | 26.35 | 9.11 | 2.74 | 10.11 | 0.79 | 0 |
| Kashmar 3 | 48.36 | 24.25 | 39.57 | 14.69 | 4.53 | 12.10 | 4.93 | 26.23 | 10.78 | 2.69 | 10.3 | 0.76 | 0 |
| Shahreza 1 | 43.20 | 23.00 | 43.76 | 19.08 | 6.52 | 9.45 | 7.76 | 11.38 | 5.67 | 0.089 | 3.22 | 0 | 1.06 |
| Shahreza 2 | 36.28 | 15.07 | 38.62 | 19.87 | 6.32 | 12.07 | 10.68 | 18.10 | 4.63 | 0.089 | 3.89 | 0 | 1.08 |
| Shahreza 3 | 32.19 | 18.16 | 34.72 | 19.57 | 6.17 | 12.72 | 10.53 | 15.77 | 3.96 | 0.078 | 3.77 | 0 | 1.02 |
| Kazerun 1 | 45.67 | 20.02 | 44.29 | 15.49 | 6.48 | 11.49 | 9.41 | 19.86 | 4.58 | 0 | 0 | 0 | 2.09 |
| Kazerun 2 | 34.46 | 19.15 | 43.35 | 15.22 | 6.20 | 13.20 | 9.90 | 16.71 | 4.72 | 0 | 0 | 0 | 2.08 |
| Kazerun 3 | 38.88 | 17.32 | 39.11 | 15.95 | 6.38 | 13.28 | 12.82 | 13.85 | 4.31 | 0 | 0 | 0 | 2.12 |
| Bojnord 1 | 58.37 | 26.30 | 28.77 | 17.88 | 5.32 | 8.48 | 8.50 | 22.17 | 6.32 | 1.81 | 0.21 | 0 | 0 |
| Bojnord 2 | 41.44 | 20.53 | 26.63 | 17.91 | 6.15 | 9.87 | 7.94 | 25.28 | 4.97 | 1.92 | 0.29 | 0 | 0 |
| Bojnord 3 | 47.10 | 23.37 | 28.21 | 17.22 | 5.43 | 9.05 | 6.82 | 28.43 | 6.18 | 1.81 | 0.24 | 0 | 0 |
| Haji Abad 1 | 51.11 | 27.26 | 39.98 | 20.50 | 5.21 | 14.50 | 11.91 | 22.50 | 7.72 | 0 | 2.78 | 0.46 | 0 |
| Haji Abad 2 | 50.73 | 22.93 | 42.92 | 19.13 | 5.99 | 14.93 | 15.56 | 22.86 | 6.43 | 0 | 2.83 | 0.44 | 0 |
| Haji Abad 3 | 57.80 | 24.57 | 40.35 | 19.53 | 5.69 | 13.02 | 14.12 | 22.65 | 7.56 | 0 | 2.96 | 0.45 | 0 |
| Sirjan 1 | 77.55 | 29.81 | 27.13 | 16.71 | 4.29 | 11.50 | 9.50 | 23.99 | 8.77 | 0.33 | 2.5 | 0.44 | 0.95 |
| Sirjan 2 | 59.47 | 23.63 | 32.37 | 16.47 | 3.61 | 12.94 | 8.73 | 23.57 | 7.51 | 0.26 | 2.69 | 0.44 | 0.96 |
| Sirjan 3 | 63.14 | 26.36 | 29.34 | 17.55 | 3.96 | 11.61 | 3.58 | 25.04 | 6.95 | 0.28 | 2.41 | 0.43 | 0.95 |
| Taft 1 | 41.71 | 21.27 | 24.76 | 19.10 | 4.09 | 16.31 | 15.85 | 29.28 | 9.14 | 0.31 | 0.57 | 0 | 0.6 |
| Taft 2 | 33.60 | 16.19 | 29.04 | 18.68 | 4.11 | 16.13 | 14.71 | 26.69 | 10.73 | 0.27 | 0.62 | 0 | 0.6 |
| Taft 3 | 34.27 | 18.51 | 27.75 | 19.49 | 4.35 | 13.74 | 14.79 | 28.20 | 8.88 | 0.26 | 0.58 | 0 | 0.61 |

**Table S2. Phenotypic values of the traits scored for the Iranian plant panel of *Glycyrrhiza* *glabra*.**

FTR: Fleshy texture of Root; CLR: Cork layer of Root

| **Population** | Fresh weight (g) | Dry weight (g) | Plant height (cm) | Plant diameter (cm) | Stem diameter (mm) | No. of branches | No. of node | Leaf length (mm) | Leaf width (mm) | Glabridin- FTR (mg/g DW) | Glabridin- CLR  (mg/g DW) | Liquritigenin- FTR  (mg/g DW) | Liquritigenin- CLR  (mg/g DW) |
| --- | --- | --- | --- | --- | --- | --- | --- | --- | --- | --- | --- | --- | --- |
| Darab 1 | 61.29 | 28.20 | 47.19 | 31.05 | 7.23 | 17.50 | 14.14 | 19.79 | 7.69 | 0 | 0.09 | 0 | 1.02 |
| Darab 2 | 46.45 | 24.77 | 49.04 | 28.96 | 6.56 | 17.13 | 11.77 | 19.68 | 6.69 | 0 | 0.05 | 0 | 1.02 |
| Darab 3 | 51.20 | 26.03 | 45.72 | 29.87 | 6.89 | 16.02 | 14.76 | 20.45 | 8.26 | 0 | 0.06 | 0 | 1.03 |
| Sepidan 1 | 66.87 | 28.34 | 41.30 | 16.54 | 5.61 | 10.70 | 11.50 | 19.15 | 8.07 | 0 | 0.04 | 0 | 0.67 |
| Sepidan 2 | 47.73 | 23.99 | 37.38 | 16.15 | 5.91 | 9.96 | 13.93 | 20.03 | 6.73 | 0 | 0.02 | 0 | 0.68 |
| Sepidan 3 | 52.27 | 25.93 | 40.28 | 15.30 | 5.77 | 11.25 | 12.82 | 17.91 | 5.16 | 0 | 0.03 | 0 | 0.68 |
| Ilam 1 | 48.48 | 23.10 | 24.91 | 14.96 | 4.09 | 7.50 | 10.40 | 22.15 | 7.67 | 0.15 | 6.65 | 1.55 | 0 |
| Ilam 2 | 31.20 | 15.29 | 27.35 | 14.98 | 4.11 | 10.13 | 9.50 | 18.07 | 6.40 | 0.14 | 6.94 | 1.53 | 0 |
| Ilam 3 | 40.89 | 17.44 | 32.29 | 14.51 | 4.35 | 9.74 | 7.67 | 16.09 | 5.30 | 0.18 | 6.87 | 1.54 | 0 |
| Semirom 1 | 52.95 | 21.06 | 27.65 | 16.14 | 5.41 | 5.50 | 9.10 | 11.88 | 6.47 | 0 | 0.09 | 0.46 | 0.87 |
| Semirom 2 | 36.01 | 19.25 | 29.56 | 16.22 | 5.54 | 7.93 | 8.44 | 15.48 | 6.91 | 0 | 0.08 | 0.47 | 0.89 |
| Semirom 3 | 44.65 | 19.38 | 26.78 | 16.01 | 5.60 | 6.41 | 6.82 | 20.02 | 7.53 | 0 | 0.1 | 0.47 | 0.9 |
| Baft 1 | 66.48 | 26.15 | 30.90 | 24.84 | 4.26 | 7.70 | 9.90 | 26.35 | 10.75 | 0.06 | 0.75 | 6.22 | 0.92 |
| Baft 2 | 52.92 | 18.05 | 33.47 | 23.16 | 4.41 | 8.88 | 8.73 | 24.15 | 9.37 | 0.04 | 0.68 | 6.23 | 0.89 |
| Baft 3 | 58.23 | 22.51 | 32.10 | 21.89 | 4.65 | 7.87 | 7.58 | 29.07 | 9.22 | 0.06 | 0.67 | 6.19 | 0.89 |
| Quchan 1 | 56.23 | 24.01 | 34.43 | 16.11 | 4.59 | 8.43 | 9.40 | 27.75 | 9.89 | 0.45 | 4.46 | 0.94 | 0 |
| Quchan 2 | 43.00 | 20.50 | 38.82 | 14.86 | 3.92 | 9.85 | 9.74 | 28.91 | 8.57 | 0.39 | 4.45 | 0.95 | 0 |
| Quchan 3 | 48.12 | 22.01 | 33.25 | 15.92 | 4.24 | 9.70 | 8.81 | 23.94 | 9.77 | 0.37 | 4.41 | 0.94 | 0 |
| Ahar 1 | 55.01 | 21.00 | 34.80 | 12.81 | 5.16 | 7.15 | 6.93 | 33.75 | 13.22 | 0.46 | 6.99 | 0 | 0 |
| Ahar 2 | 39.95 | 17.35 | 27.98 | 12.67 | 5.30 | 5.62 | 10.25 | 31.18 | 10.17 | 0.48 | 6.88 | 0 | 0 |
| Ahar 3 | 48.53 | 19.03 | 30.42 | 12.54 | 5.35 | 8.49 | 8.74 | 38.16 | 11.87 | 0.42 | 6.85 | 0 | 0 |
| Bardsir 1 | 63.91 | 26.61 | 44.25 | 14.69 | 6.28 | 11.50 | 8.25 | 20.98 | 8.28 | 0 | 0.95 | 4.41 | 0.73 |
| Bardsir 2 | 46.23 | 21.93 | 45.01 | 14.95 | 5.58 | 10.93 | 9.04 | 23.47 | 7.44 | 0 | 0.9 | 4.37 | 0.73 |
| Bardsir 3 | 54.28 | 23.64 | 42.87 | 14.80 | 6.52 | 12.25 | 10.53 | 24.33 | 6.31 | 0 | 0.88 | 4.36 | 0.72 |
| Eghlid 1 | 73.16 | 36.46 | 24.89 | 16.94 | 6.21 | 9.84 | 5.89 | 23.03 | 10.82 | 0 | 0.36 | 0 | 0.49 |
| Eghlid 2 | 54.33 | 24.42 | 27.28 | 17.05 | 6.99 | 13.49 | 7.12 | 20.89 | 9.07 | 0 | 0.35 | 0 | 0.51 |
| Eghlid 3 | 62.69 | 30.04 | 27.44 | 17.27 | 6.69 | 10.98 | 8.42 | 20.95 | 7.00 | 0 | 0.41 | 0 | 0.47 |
| Soltanieh 1 | 39.82 | 16.03 | 26.75 | 12.30 | 4.59 | 8.50 | 6.50 | 22.31 | 11.65 | 0.05 | 0.21 | 0.75 | 0.54 |
| Soltanieh 2 | 30.43 | 11.85 | 29.57 | 12.00 | 3.92 | 7.94 | 8.93 | 23.83 | 9.58 | 0.07 | 0.27 | 0.76 | 0.56 |
| Soltanieh 3 | 35.00 | 13.67 | 26.91 | 12.30 | 4.24 | 6.82 | 5.82 | 24.10 | 11.65 | 0.04 | 0.23 | 0.72 | 0.53 |
| Saqez 1 | 43.88 | 14.77 | 23.67 | 13.10 | 4.83 | 9.25 | 9.50 | 13.09 | 4.12 | 0 | 1.14 | 0 | 0.32 |
| Saqez 2 | 30.93 | 12.16 | 26.33 | 13.00 | 4.00 | 11.99 | 8.73 | 11.35 | 4.38 | 0 | 1.26 | 0 | 0.31 |
| Saqez 3 | 36.88 | 13.37 | 27.14 | 13.20 | 4.39 | 10.42 | 11.58 | 11.50 | 4.31 | 0 | 1.21 | 0 | 0.32 |

FTR: Fleshy texture of Root; CLR: Cork layer of Root
